# Prioritising access to pandemic influenza vaccine: a review of the ethics literature

**DOI:** 10.1186/s12910-020-00477-3

**Published:** 2020-05-14

**Authors:** Jane H. Williams, Angus Dawson

**Affiliations:** 1 Sydney Health Ethics, Sydney School of Public Health, University of Sydney, Level 1, Medical Foundation Building K25, Sydney, NSW 2006 Australia; 2 Marie Bashir Institute for Infectious Disease and Biosecurity, University of Sydney, Sydney, Australia

**Keywords:** Ethics, Pandemic influenza, Vaccine, Prioritisation, Critical interpretative review

## Abstract

**Background:**

The world is threatened by future pandemics. Vaccines can play a key role in preventing harm, but there will inevitably be shortages because there is no possibility of advance stockpiling. We therefore need some method of prioritising access.

**Main text:**

This paper reports a critical interpretative review of the published literature that discusses ethical arguments used to justify how we could prioritise vaccine during an influenza pandemic. We found that the focus of the literature was often on proposing different groups as priorities (e.g. those with pre-existing health conditions, the young, the old, health care workers etc.). Different reasons were often suggested as a means of justifying such priority groupings (e.g. appeal to best overall outcomes, fairness, belonging to a vulnerable or ‘at risk’ group etc.). We suggest that much of the literature, wrongly, assumes that we are able to plan priority groups prior to the time of a particular pandemic and development of a particular vaccine. We also point out the surprising absence of various issues from the literature (e.g. how vaccines fit within overall pandemic planning, a lack of specificity about place, issues of global justice etc.).

**Conclusions:**

The literature proposes a wide range of ways to prioritise vaccines, focusing on different groups and ‘principles’. Any plan to use pandemic vaccine must provide justifications for its prioritisation. The focus of this review was influenza pandemic vaccines, but lessons can be learnt for future allocations of coronavirus vaccine, if one becomes available.

## Background

A human influenza pandemic occurs when a novel virus, usually originating in birds or animals, starts circulating in a population with limited or no underlying immunity. As a result, the virus can transmit more easily and potentially more seriously affect those infected. Pandemic influenza is distinct from seasonal influenza, in that it may strike at any point in the year, its clinical features are less predictable and it may disproportionately affect population groups who are not generally at high risk of harm from seasonal influenza.

When an influenza pandemic strikes, there will be an opportunity to develop a number of preventive and treatment options. One extremely important decision is to trigger the manufacture and then distribute a vaccine to prevent infection. However, because the virus is novel, it is not possible to create and stockpile pandemic vaccine in advance. The World Health Organization (WHO) estimates that it may take five to six months, from the first reports of infection, before the first batches of vaccine are available for use. Once vaccine becomes available, there will be insufficient vaccine at that time for everyone that may want it due to limited manufacturing output. Vaccine limitation is therefore inevitable in the early stages of an influenza pandemic.

The 2009 H1N1 influenza pandemic highlighted shortcomings in national and WHO preparedness and response. In the aftermath of the pandemic and criticisms of how it was managed, WHO developed a pandemic influenza preparedness framework [1] and urged national agencies to create plans, or to update existing plans, accordingly. This work is ongoing. Many, but not all, countries have strategies in place that cover a wide range of pandemic preparedness considerations. One of these is how to plan for and manage limited vaccine availability in the population. This aim is often structured in terms of producing a list of identifiable groups in a population that is prepared in advance [2]. However, planning an ordered list of priority groups for access to limited vaccine is not a simple, or arguably desirable, matter as there will always be a number of relevant uncertainties about both the nature of the pandemic and the vaccine. For example, the general clinical severity of the pandemic virus is unpredictable. Which sub-groups in a population, if any, are at greater risk will not be apparent until some time after the outbreak hits, and sufficient surveillance data is collected. Time pressures mean that vaccine efficacy and safety data in different populations will be available only after the vaccine is in widespread use. Difficulty notwithstanding, preparedness planning that includes prioritising certain population groups in pandemics is recommended. Planning is best done in advance of an emergency, although any plan can only be a starting point, and will always need to be flexible and adapted once we have more information. As we are currently in an interpandemic phase it is an optimum time to take stock of previous planning attempts and pandemic experiences and do what we can to prepare for the next, inevitable influenza pandemic. This review is an attempt to provide a starting point for those working in countries, such as Australia, where pandemic vaccine priority plans are being developed or updated.

There is a reasonably large literature about ethics and pandemics. It can be broadly characterised as framing discussions around the legitimacy of restricting liberties or interfering with the property rights of individuals [3, 4]. The common assumption seems to be that the most ethically contentious aspects of action in response to a pandemic will require people to change their behaviour in various ways (e.g. accepting the need for quarantine and social distancing or requiring their property to be requisitioned etc.). This review had a narrower focus to reflect the specific question: what ethical arguments have been used to justify different approaches to prioritising vaccine access during an influenza pandemic? The values of liberty and autonomy that dominate the literature are less likely to be relevant to this question. Vaccine rationing does not involve restricting liberties, but rather deciding who to vaccinate first in a situation where many may want vaccination. It is therefore more of a question appealing to discussions of just distributions and deciding what matters and who matters when deciding how to respond. In this paper we present a critical interpretative synthesis of the literature and discuss its relevance for the Australian context.

## Main text

We conducted a critical interpretive review (CIR) of the ethics literature, drawing on Ros McDougall’s extension of Dixon-Woods et al’s method of critical interpretive synthesis [5, 6]. A critical interpretive review is not a traditional systematic review of the literature that seeks to aggregate evidence about the impact of an intervention. It is rather a method that seeks to capture the *key ideas* relevant to a particular issue and was chosen for its applicability to questions that examine the ethics literature. This method has a number of advantages over traditional systematic reviews when addressing normative questions. For example, terms used in ethical discussion can be used vaguely and inconsistently in the literature and they tend to be poorly tagged in databases. Our experience suggests that systematic searches in relation to ethics terms are likely both to return search results that are not relevant to the topic while at the same time excluding pertinent literature. A CIR allows the incorporation of a number of different search methods in the same review, compensating for a difficulty in aggregating results from this type of literature. For this review we employed six varied search techniques between April and August 2018. As an initial step, one of the authors (AD) has had a longstanding interest in the topic and identified relevant papers that were already known to him. To this group, we added papers retrieved from searches of Google Scholar, Phil Papers, and Medline. Search terms were variations on ‘pandemic + vaccinations + ethics’, plus or minus ‘influenza’, ‘priority’ and/or ‘rationing’, adapted to suit the database. As a third step, we conducted a content search of the top 20 bioethics journals according to a metric generated by Google Scholar in August 2018. Finally, we checked and followed up on the references in the literature we had identified as relevant. The combination of these search strategies returned 61 papers after title and abstract checking. JW then read the full text to check relevance against our inclusion criteria. These criteria were: published in English, peer reviewed, and contained either a normative argument about who or what to prioritise or how we should allocate pandemic influenza vaccine or commentary on others’ normative arguments. There was no date restriction. After review of the full text revision, 40 papers were included. See the appendix for the list.

We did not include grey literature in this review, though some of it is reported in included peer reviewed papers. While some national bodies have published reports that outline how to prioritise different groups for vaccination in a pandemic the majority did not directly address ethical reasons or contain substantive normative arguments in those documents [7]. An exception is the robust ethical reasoning underpinning New Zealand’s planned pandemic response [8]. We direct interested readers to existing reviews of aspects of the grey literature on pandemic planning [9–13].

We read the literature with a view to identifying the breadth of normative claims about who or what to prioritise but also to analyse it as a whole. Critical interpretive reviews call not only for the identification and description of key ideas in answering a question but also for their interpretation. The review was therefore a two-step process: pulling normative arguments from individual papers; and subsequently synthesising the body of literature to gain an understanding of similarities, differences, and omissions. We present this review in the same way: first with a descriptive presentation of the literature followed by a critical analysis and discussion of key issues.

## Results

The literature overwhelmingly originates in North America and Europe and tends to assume the conditions of a liberal western democracy: an existing well-run and well-funded immunisation programme, and a transparent and well-resourced health system that can support the planning and implementation of pandemic immunisation. While we did not restrict dates in our searches, the majority of the literature was published in 2006–2009 and 39 of the 40 papers were published between 2005 and 2015. Whether or not it was explicitly acknowledged, the pandemics of 2003–4 (SARS) and 2009 (H1N1) appear to have driven interest in the ethical issues surrounding the rationing of pandemic influenza vaccine. The literature contained a mix of contexts; some were more speculative and theoretical, others drew on specific experiences. While a minority of the papers did not differentiate between seasonal and pandemic influenza vaccine planning, most were explicit about the importance of differentiating between the two cases, and some noted that basing access to pandemic vaccine on seasonal flu planning was unjustifiable.

Below we describe what the literature says about who, what, and which values matter in prioritising pandemic vaccine access.

### Who matters: a large number of diverse priority groupings has been proposed

There is a wide variety of suggested populations for possible priority access to vaccine in the literature. Different papers took different approaches to how to prioritise different populations. While some ranked groups in order of importance, many did not. Others made normative arguments that might be used to rank groups, but did not actually specify any priority populations. Different kinds of groupings are reflected in the discussions, including those related to occupations, stage of life, health status and social status. Some literature rejected the very idea of specifying groups and instead proposed different procedures held to be ‘fair’ for the whole population.

The most commonly justified group given priority was healthcare workers (HCW) [14–25], though the parameters around the nature and size of this group varied based on professional status and proximity to pandemic-affected patients. Other occupational groups for priority access included vaccine manufacturers, emergency services workers, and those working in basic infrastructure such as utility, transport, policing, food manufacturing and distribution, and communications [15, 16, 18, 19, 21, 23, 25, 26]. Stage of life arguments were also made. Children were prioritised by some [14, 22–24, 27–29], either as a means to minimise infection in the community or because they had the longest lives ahead of them. A number of papers argued for priority access for an age cohort running from adolescence to pre-middle aged adults for a number of different reasons (e.g. an appeal to ‘fair innings’ arguments, the contribution that this cohort can make in terms of future collective benefit through the use of their existing skills etc.) [25, 28–31]. Some made the case for the sickest or the most medically vulnerable to receive priority vaccination [15, 16, 23, 25, 26, 32–34], usually framed in terms of ‘high risk’ of harm, although they differed on whether medical need was about susceptibility to pandemic infection or poor baseline health. Others justified priorities based on social vulnerability [3, 14, 35] and included, variously, homeless people, ‘hard to reach’ groups, and socially stigmatised groups such as prisoners and obese people. It is worth noting here that the concept of vulnerability was used frequently, often as a catch-all, but regularly not defined or explained. Some of the literature took a different tack, arguing for vaccinating in social clusters where individuals were not associated with demographic or health-related commonalities but rather by social connection, such as extended family groupings [3, 16, 36]. Finally, some argued for priority access to be granted on the basis of a set procedure for the whole population, for example via a lottery, rather than on the basis of setting priority groups in advance [20, 29, 33, 37–39]. Lotteries were also rejected by others [16, 40].

The groups described above all had points in common. They were groups of people who were young, or ill, or worked in a particular job. This reflects a fairly consistent conceptualisation in the literature of thinking of population groups as collections of individuals with different kinds of demographic characteristics. Sometimes such groupings were treated as if they reflected the only feature that mattered, in other papers it was acknowledged that some of these characteristics might be combined, giving two or more reasons to prioritise some individuals. For example, a person might fit in a more ‘at risk’ age category, but also have an underlying health condition that also increased risk, and perhaps be homeless. Is such a person a priority or are they a priority multiplied by three? The answer, in the literature, was unclear.

### What matters: the aims of pandemic vaccination programs

As with the variety of groupings in the literature, there was a wide spread of articulated aims underpinning them. By far the most commonly articulated goal of pandemic vaccination was to prevent (the most) illness [16, 26, 41] and/or save (the most) lives [23, 24, 27, 29, 32, 33]. This was framed in a variety of ways: benefitting greatest number of individual people [34]; maximising Quality of Life Years Saved (QALYS) or minimising years of life lost (YLL) [26, 30]; saving the worst off [35]; saving those most likely to recover [29]; saving younger lives [22, 28, 29]; saving those most likely to contribute to a flourishing society (either economically or socially) [31], and; saving those who can most usefully contribute to minimising the impact of the pandemic [17, 22].

A minority of papers also expressed aims for the vaccination programme that were framed differently. Some focused not on who became infected and ill but rather who was more likely to play a significant role in spreading infections, with the aim of reducing this as much as possible [14]. For example, we might target school children for priority vaccination, less for their direct benefit than as a means to reduce transmission of the virus in the population. Others did not focus on the physiological effects of pandemic influenza virus at all. These papers made a plea for overall societal benefits, such as maintaining social order [15, 18, 19], or emphasised what they understood to be the core work of public health ethics: promoting justice, solidarity and trust in government and public health systems, especially when these are likely to come under threat (as in a pandemic) [3, 20, 23, 35, 36, 38, 42–44].

As previously mentioned, a hallmark of vaccinating against pandemic influenza is the degree to which decisions will need to be made in conditions of uncertainty. As such many, but not all, of the papers in this review described the scenarios under which their arguments about who and/or what mattered would hold or they noted that who and what mattered could change depending on the severity of the pandemic [25]. Changing conditions, therefore, could lead to changing priorities. For example, an aim of preventing the most illness could be justified initially and for a mild pandemic. In the event of a severe pandemic, however, maintaining social order was considered increasingly important. In that case, priority populations for vaccine access would change as the aims for the vaccination program changed, and those aims would change mostly in response to the perceived severity of the outbreak.

Finally, in acknowledgment of the difficulty inherent in managing goals that may not be commensurate with each other, some papers focused on the primary importance of procedural ethics in making and communicating decisions about who to prioritise for pandemic vaccine access [25, 26, 44, 45].

### Justifying who and what to prioritise: the ethics arguments

The literature was diverse in its treatment of the ethical underpinnings of normative recommendations for allocating limited vaccine in a pandemic. Some of it drew on experiences of previous pandemics and was highly applied (e.g. [41, 45]), other papers were more abstract and theory-driven (e.g. [3, 36]). As such there was varied emphasis across the different papers on the role of normative argumentation as such in justifying what or who mattered in the case of priority access to pandemic vaccine. There was a tendency in some of the literature for authors to list principles or values for consideration in vaccine rationing, without necessarily explaining how they might be used in practice in response to a particular scenario or even whether the listed values were complementary or even reconcilable with each other.

Most of the literature took a line that was broadly either explicitly or implicitly consequentialist in nature, with a tendency to be focused on outcomes, with appeal to a good to be maximised or a harm to be minimised (such as greatest number of survivors, least illness or fewest deaths). Justifications were not usually strictly utilitarian. While they took positions on how to allocate vaccine that maximised health outcomes, papers tended to advocate for other non-utilitarian values (such as procedural justice) to be considered in achieving those outcomes. Maximising aggregate population health was used to argue for prioritising HCW, on the understanding that healthy HCW would be available to care for the ill and thus minimise morbidity and mortality. It was also used to support vaccinating those with highest medical need in order to reduce overall health-related harm. Others used the same outcome-focused reasoning to prioritise not those who would get ill but rather those who had the most potential to make others ill, arguing variously for the vaccination of children, prisoners, homeless people – all groups for whom social distancing might be difficult. There were also some more classically egalitarian arguments in the literature justifying lotteries (giving every equally-valuable individual an equal chance of benefiting) and ‘first come first served’ systems that were supported and rejected on the grounds of both fairness and efficiency.

Many of the papers included in this review appealed to at least one version of justice (or fairness, or equity, or similar). This set of concepts is often poorly defined in the public health ethics literature when not the central focus of a paper, and the vaccine prioritisation literature is, for the most part, not an exception. Distributive justice arguments tended to support giving priority to ‘vulnerable’ groups but the vulnerability in question was not always defined. Equity arguments were sometimes used to support equal treatment of individuals and sometimes to justify prioritising the needs of those groups held to be worse off. On the other hand, procedural justice was well-described and usually followed an ‘Accountability for Reasonableness’ (A4R) model [46]. Its central place in pandemic vaccine planning was mostly justified by the importance of trust in government and health systems during a pandemic. Most argued for procedural justice alongside substantive ethical considerations but some made the point that an advantage of A4R is that it supposedly permits decision-making in the absence of agreement on principles and priority groupings.

Other ethical justifications for judgments about prioritising were made. Reciprocity was a second key driver for allocating priority vaccine to HCW, particularly in the literature that reported on or responded to the experience of SARS. These papers argued that if HCW were considered to have a special obligation to attend work in times of increased personal risk then they (and perhaps their families) ought to be recompensed in the form of priority access to vaccine. Finally, a minority of papers took a more collective, solidaristic approach to the ethics of vaccine rationing. One argued that public health agencies must consider both the “expressive function” of public health and the underlying message(s) and social meanings generated by pandemic planning and the creation of (often arbitrary) social groupings [36]. Another advocated for a relational approach to pandemic planning, that is, one that situates preparations and response to a pandemic within the broader project of promoting public health and justice [3].

## Discussion

### The pressure to (over)plan

Many of the papers included in this review opened with an acknowledgement that health authorities were encouraged or required to plan for future pandemics, including the provision of priorities for the allocation of limited vaccine. Planning is of course important. However, depending on the goal of the vaccination programme, it is likely that evidence about the behaviour of both the particular pandemic virus (which groups may be at most risk of harm, patterns of spread) and the vaccine (effectiveness and possible side-effects, whether some population groups are better or worse protected, and the number of doses required) will be needed before final decisions can be made about priorities. If the primary or only goal of vaccinating is to reduce morbidity and mortality in the population, proposing certain priority groups in advance of the pandemic could in the best case give public health agencies an effective head start. However, in the worst, and probably more likely, case having predetermined groups could contribute to vaccine wastage or even frustrate other possible aims for a programme. In the event that the goal of the vaccination program is to promote confidence and trust, it may be that predetermined priority groups are more appropriate. This is because making decisions about this in advance allows public health agencies to communicate simpler messages to the public, who may experience enhanced trust because they know what to expect. However, a particular pandemic may be more or less severe than anticipated, and its toll on people and society may occur in ways that are unexpected. In those scenarios, it may be that the goals of a vaccination programme may need to be flexible and changed as evidence is obtained from surveillance and other systems.

### Thinking normatively about vaccine scarcity in relation to other pandemic mitigation measures

Vaccination is just one element of a comprehensive response to a pandemic. Other measures, depending on severity, may include the provision of (possibly limited) prophylactic medications, social distancing, voluntary or enforced quarantine of those who might have been exposed, isolation of those infected, and border closures. The normative literature did not consider vaccine rationing as part of such an overall pandemic plan. While it is complex and difficult, we think it would be more useful to assess vaccine priorities in conjunction with such other possible measures. For example, vaccine status might have the potential to impact upon the freedom of movement of individuals in the event that non-vaccinated individuals are judged to be at higher risk of being infected and thereby infecting others. Or it might be important to consider whether it is fair for the same groups of people (e.g. those at highest medical risk) to have priority access to both vaccine and anti-viral medication. Or whether those who can be socially isolated (e.g. the elderly) should be, and whether ease of social isolation affords them lower vaccine priority. There is little discussion about how vaccine priority issues relate to these other important trade-offs.

### Clarity in language: shared assumptions or are we using the same words to talk about different things?

It was common for subpopulations to be referred to as ‘vulnerable’, and sometimes as ‘high risk’. There was often little clarity about what the groups in question were vulnerable to or at risk of – whether particular aspects of the pandemic in question meant they were at greater risk of harm than others in the population, or whether there were pre-existing factors relating to their health status that meant they were at greater risk. There are, no doubt, other possible explanations for how vulnerability and high risk were used, but the broader point is that these labels act as motivating descriptors, presumably intended to imply certain group characteristics that require us to respond to them. Littman discusses the imprecision of the use of risk in pandemic vaccine prioritisation, and calls attention to the definitions and thresholds that are (not) used to influence vaccine access [47]. We suggest that further substantive work about how these categories are defined, used and applied is warranted.

### Planning for different experiences of the same pandemic

The literature is overwhelmingly North American and Northern European in its origin, perhaps not a surprise given our restrictions to English language papers. However, this potentially masks a more telling observation, which is that much of the literature discusses the issue of vaccine prioritisation as though it is irrelevant where we are situated. Its consistency in ignoring potential differences in healthcare systems, social experience and value systems seems to inadvertently contribute to the idea that we can respond to such questions as if viewing from nowhere. Both of the reviewers are based in Australia and noted that ethical considerations that are relevant to the Australian context are not found in the normative literature on prioritising pandemic influenza vaccine. We raise two such considerations here, and make the broader point that each country is likely to have its own particular ethical concerns. These need to be made visible and discussed.

Australia is a vast country and has unique geographical considerations. The bulk of the population is concentrated in cities along the coasts but there are also many regional and remote communities with distinctive characteristics and needs. Geographical distinctions did not feature in the literature we reviewed, but may raise significant questions about vaccine access. For example, in some countries, whilst there may be no intention to prioritise an urban over a rural population, the realities of existing imperfect logistical systems may result in unequal distribution and thereby access. Other things being equal it is easier and quicker to deliver vaccine in urban areas, this then potentially aids the efficient use of vaccine. But rural populations may have less access to health care facilities, so we might see them as another vulnerable or at risk group. Perhaps we have reason to think that rural populations are only likely to come into contact with the virus later in a pandemic? Such a claim will require us to have robust evidence to back it up. Otherwise, this is something that we should take into account in our planning. How do we establish a set of priorities for a pandemic vaccine that do not unintentionally contribute to unfairness in the population?

The literature assumes that the relevant parameters of the discussion of vaccine prioritisation operate within the borders of sovereign nations. However, is it obvious that this is a relevant limit to our discussions? Australia, for example, clearly has obligations to its less well-resourced neighbours. What exactly follows from those obligations will be disputed, but we should not just simply assume that vaccine prioritisation means that we should always focus on our ‘home’ population. Or at least we should not do so in the absence of compelling reason why we should. As discussion of vaccine distribution is often formulated in terms of prioritarian concerns, focused on groups seen to be vulnerable or at greater risk, if groups in neighbouring countries are at greater threat of harm, can we justify ignoring a parallel claim from them upon us? These are complex issues and we do not discuss or defend cosmopolitanism here, but it is interested that one major omission in the literature about pandemic vaccine prioritisation is the lack of attention to global distributive concerns. This is particularly striking given the limited number of countries with the capacity to manufacture vaccine in response to a pandemic. Country of residence might turn out to be a significant contributing factor to the chances of any individual receiving vaccine. To what degree, if any, is this a relevant factor in a fair distribution of vaccine? There has been some attempt to address this policy issue by the WHO through a proposed Pandemic Influenza Preparedness (PIP) framework and related material transfer agreements [48]. However, it is unclear how such an approach may fair during an actual pandemic.

## Conclusion

We conducted a critical interpretive review of the normative literature about how limited pandemic vaccine should be prioritised. Recommendations varied, though the majority of the normative arguments put forward in the literature could be characterised as broadly consequentialist. Most of the papers also called attention to a version of justice, although this was often quite vague. Further applied work in ethics can contribute to the clarification of possible justifications for different distributions of pandemic vaccine and how these issues relate to pandemic planning in general. The literature does not draw on how particular contexts (e.g. healthcare system, funding, population distribution) might call for different approaches to prioritisation. Attention to context in normative arguments, and not assuming a uniform pandemic experience, would generate a richer literature that could help inform nuanced and careful approaches to planning. Differences in context may be national, regional or population-based and we would encourage applied ethicists to be specific about the socio-political situation that underlies the normative argument being made. In addition, the literature does not address how vaccine access might interact with other pandemic protection measures. How might arguments for prioritising different groups look in the context of access to other preventive measures? If an individual receives a vaccine, does she have less obligation to distance herself from others? Does someone who was not prioritised for limited vaccine access have a greater claim to other limited health resources if they become very ill? Addressing this type of question could further develop normative arguments about prioritising pandemic vaccine by placing it in the broader context of pandemic prevention and care. Finally, this review has highlighted the imprecise use of both ethics ideas (equity, fairness) and concepts central to thinking about pandemics (risk, vulnerability). Further clarification of these concepts and their judicious application will contribute to arguments about prioritising limited resources in a pandemic. The focus of this review was influenza pandemic vaccines. However, some of the issues, concepts and arguments will prove useful in considering the allocation of future coronavirus vaccine, assuming that one becomes available.

## Supplementary information

**Additional file 1.** Appendix: Papers Included in Review.

## Data Availability

All data generated or analysed during this study are included in this published article and its appendix.
