# Supplementary material for: Prioritising access to pandemic influenza vaccine: a review of the ethics literature
Source: BMC Med Ethics. 2020 May 14;21:40. doi: 10.1186/s12910-020-00477-3 (PMC7224123; doi:10.1186/s12910-020-00477-3)
Supplement: Supplementary file 1 — Additional file 1. Appendix: Papers Included in Review. [file 12910_2020_477_MOESM1_ESM.docx]

# Appendix: Papers included in review

Arras J. Rationing Vaccine During an Avian Influenza Pandemic: Why It Won't Be Easy. Yale Journal of Biology and Medicine. 2005;78:283-96.

Bambery B, Douglas T, Selgelid MJ, Maslen H, Giubilini A, Pollard AJ, et al. Influenza Vaccination Strategies Should Target Children. Public Health Ethics. 2017.

Baylis F, Kenny NP, Sherwin S. A Relational Account of Public Health Ethics. Public Health Ethics. 2008;1(3):196-209.

Berkman BE. Incorporating explicit ethical reasoning into pandemic influenza policies. Journal of Contemporary Health Law and Policy. 2009;26(1):1-19.

Bhatia P. The H1N1 influenza pandemic: need for solutions to ethical problems. Indian Journal of Medical Ethics. 2013;X(4):259-63.

Blumenshine P, Reingold A, Egeter S, Mockenhaupt R, Braveman P, Marks J. Pandemic Influenza Planning in the United States from a Health Disparities Perspective. Emerging Infectious Diseases. 2008;14(5):709-15.

Buccieri K, Gaetz S. Ethical Vaccine Distribution Planning for Pandemic Influenza: Prioritizing Homeless and Hard-to-Reach Populations. Public Health Ethics. 2013;6(2):185-96.

Devnani M, Gupta AK, Devnani B. Planning and response to the influenza A (H1N1) pandemic: ethics, equity and justice. Indian Journal of Medical Ethics. 2011;4(Oct-Dec):237-40.

Draper H, Sorell T, Ives J, Damery S, Greenfield S, Parry J, et al. Non-Professional Healthcare Workers and Ethical Obligations to Work during Pandemic Influenza. Public Health Ethics. 2009;3(1):23-34.

Emanuel E, Wertheimer A. Who Should Get Influenza Vaccine When Not All Can? Science. 2006;312(5775):854-5.

French PE, Raymond ES. Pandemic Influenza Planning: An Extraordinary Ethical Dilemma for Local Government Officials. Public Administration Review. 2009;69(5):823-30.

Gostin L. Medical Countermeasures for Pandemic Influenza: Ethics and the Law. JAMA. 2006;295(5):554-6.

Hirose I. Should we select people randomly? Bioethics. 2010;24(1):45-6.

Kaposy C, Bandrauk N. Prioritizing Vaccine Access for Vulnerable but Stigmatized Groups. Public Health Ethics. 2012;5(3):283-95.

Kass NE, Otto J, O'Brien D, Minson M. Ethics and severe pandemic influenza: maintaining essential functions through a fair and considered response. Biosecur Bioterror. 2008;6(3):227-36.

Kayman H, Ablorh-Odjidja A. Revisiting Public Health Preparedness: Incorporating Social Justice Principles Into Pandemic Preparedness Planning for Influenza. Journal of Public Health Management and Practice. 2006;12(4):373-80.

Kinlaw K, Barrett DH, Levine RJ. Ethical guidelines in pandemic influenza: recommendations of the Ethics Subcommittee of the Advisory Committee of the Director, Centers for Disease Control and Prevention. Disaster Med Public Health Prep. 2009;3 Suppl 2:S185-92.

Kotalik J. Preparing for an Influenza Pandemic: Ethical Issues. 19. 2005;4(422-431).

Lee C, Rogers WA, Braunack-Mayer A. Social Justice and Pandemic Influenza Planning: The Role of Communication Strategies. Public Health Ethics. 2008;1(3):223-34.

Littman J. Distributing Vaccine Fairly During Influenza Pandemics - A Case Study from Berlin. 2013. In: Ethics in Public Health and Health Policy: Concepts, Methods, Case Studies [Internet]. Springer; [1175-192].

Littmann J. How high is a high risk? Prioritising high-risk individuals in an influenza pandemic. Vaccine. 2014;32(52):7167-70.

McLachlan HV. A proposed non-consequentialist policy for the ethical distribution of scarce vaccination in the face of an influenza pandemic. J Med Ethics. 2012;38(5):317-8.

McLachlan HV. On the random distribution of scarce doses of vaccine in response to the threat of an influenza pandemic: a response to Wardrope. Journal of Medical Ethics. 2015;41(2):191-4.

Miller MA, Viboud C, Olson DR, Grais RF, Rabaa MA, Simonsen L. Prioritization of influenza pandemic vaccination to minimize years of life lost. J Infect Dis. 2008;198(3):305-11.

Persad G, Wertheimer A, Emanuel E. Principles for allocation of scarce medical interventions. The Lancet. 2009;373:423-31.

Peterson M. The moral importance of selecting people randomly. Bioethics. 2008;22(6):321-7.

Peterson M. Pandemic influenza and utilitarianism. Bioethics. 2011;25(5):290-1.

Pierce R. The Expressive Function of Public Health Policy: The Case of Pandemic Planning. Public Health Ethics. 2011;4(1):53-62.

Rebmann T, Zelicoff A. Vaccination against influenza: role and limitations in pandemic intervention plans. Expert Rev Vaccines. 2012;11(8):1009-19.

Riley S, Wu JT, Leung GM. Optimizing the dose of pre-pandemic influenza vaccines to reduce the infection attack rate. PLoS Med. 2007;4(6):e218.

Rothstein MA. Currents in Contemporary Ethics: Should Health Care Providers Get Treatment Priority in an Influenza Pandemic? Journal of Law, Medicine & Ethics. 2010;38:412-9.

Schwartz B, Orenstein WA. Prioritization of pandemic influenza vaccine: rationale and strategy for decision making. Curr Top Microbiol Immunol. 2009;333:495-507.

Selgelid MJ. Pandethics. Public Health. 2009;123(3):255-9.

Sheather J. Ethics in the face of uncertainty: preparing for pandemic flu. Clinical Ethics. 2006;1(4):224-7.

Thompson AK, Faith K, Gibson JL, Upshur RE. Pandemic influenza preparedness: an ethical framework to guide decision-making. BMC Med Ethics. 2006;7:E12.

Vawter DE, Gervais KG, Garrett JE, Pandemic Influenza Ethics Work G. Allocating pandemic influenza vaccines in Minnesota: recommendations of the Pandemic Influenza Ethics Work Group. Vaccine. 2007;25(35):6522-36.

Verweij M. Moral Principles for Allocating Scarce Medical Resources in an Influenza Pandemic. Journal of Bioethical Inquiry. 2009;6(2):159-69.

Wardrope A. Scarce vaccine supplies in an influenza pandemic should not be distributed randomly: reply to McLachlan. J Med Ethics. 2012;38(12):765-7.

Wynia MK. Ethics and public health emergencies: rationing vaccines. Am J Bioeth. 2006;6(6):4-7.

Zimmerman RK. Rationing of influenza vaccine during a pandemic: ethical analyses. Vaccine. 2007;25(11):2019-26.
